# BRCA2 mutations in familial breast cancer with prostate cancer: a case report and literature review

**DOI:** 10.3389/fonc.2024.1428849

**Published:** 2024-09-19

**Authors:** Zhengsheng Liu, Qianhao Huang, Meixuan Ding, Tao Wang, Yuedong Chen, Kaiyan Zhang

**Affiliations:** ^1^ The Key Laboratory of Urinary Tract Tumors and Calculi, Department of Urology, The First Affiliated Hospital of Xiamen University, School of Medicine, Xiamen University, Xiamen, China; ^2^ The School of Clinical Medicine, Fujian Medical University, Fuzhou, China

**Keywords:** BRCA2 mutations, BRCA2, familial breast cancer, familial prostate cancer, prostate cancer

## Abstract

Prostate cancer (PCa) is the second most common tumor in men globally. Its etiology has been attributed to multiple factors, including age and ethnicity, with family history identified as a significant risk factor. The role of family history in prostate cancer risk appears to be more extensive than previously thought, with evidence suggesting that prostate cancer and breast cancer may occur concurrently within families. BRCA2 mutations have been linked to an increased risk of prostate cancer, particularly in patients diagnosed with early-onset disease. It is estimated that BRCA2 mutations account for approximately 5% of familial prostate cancer cases. It is noteworthy that cases of prostate cancer in patients with BRCA2 mutations are rare in clinical practice. Here we report a case of prostatitis carcinoma with a mutation in the BRCA2 gene in a patient who underwent robotic-assisted radical prostatectomy for prostatitis carcinoma after medication was not effective. Genetic testing of him, his son, and his daughter showed that they all had mutations in this gene, and it is noteworthy that the type of BRCA2 mutation in his son has never been reported before, which is rare in clinical practice.

## Graphical Abstract

Patient Course at a Glance.

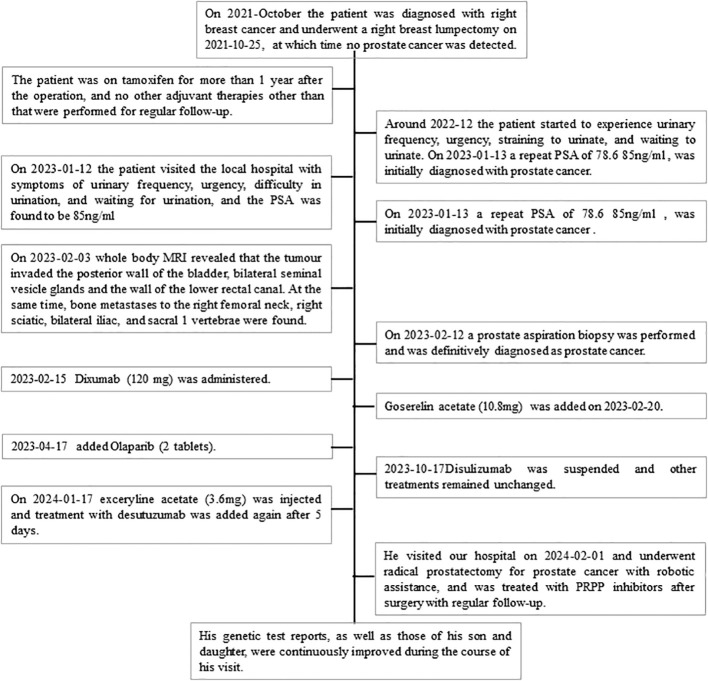

## Introduction

Prostate cancer (PCa) is the second most common tumor in men globally, with about 900,000 new cases appearing each year, despite wide variations in incidence in different regions. Its etiology is related to a number of factors, including age and race, and family history is considered an important risk factor. About 20% of prostate cancer cases have a familial clustering,BRCA2 mutations in only 5% of PC cases (1) The role of family history in prostate cancer risk appears to be more widespread than previously thought, and there is evidence that prostate and breast cancer can occur together in some patients. The hereditary breast cancer-related gene 2 (BRCA2) has been identified as a key contributor to these conditions. The contribution of BRCA1 and BRCA2 to cancer has been extensively studied, as families with these mutations show clustering of cancers in men. These genes are not only associated with an increased risk of developing breast, ovarian, fallopian tube and peritoneal cancers in women. They are also associated with having male breast and prostate cancers (1, 2). BRCA2 is estimated to be present in about 1-2% of sporadic prostate cancer cases, leading to an 8.6-fold increase in the risk of prostate cancer, while BRCA1 increases the risk of sporadic prostate cancer by 3.5-fold, but it is present in only 0.44% of prostate cancer cases (2, 3).

BRCA1 and BRCA2 are tumor suppressor genes, both of which are inherited in an autosomal dominant manner with incomplete epistasis. Tumorigenesis in individuals with germline mutations in the BRCA gene requires somatic inactivation of the remaining wild-type allele. Both genes encode large proteins that function in multiple cellular pathways. loss of function of either BRCA1 or BRCA2 is associated with defects in the repair of DNA double-strand breaks (DSBs) through the conserved mechanism of homologous recombination (HR). Therefore, cells must repair these lesions by other non-conservative and potentially mutagenic mechanisms. This genomic instability may underlie cancer susceptibility caused by deleterious mutations in the BRCA gene. On the clinical side, several epidemiological studies have examined the risk of prostate cancer in BRCA1 and BRCA2 mutation carriers, and BRCA2 mutations increase the risk of prostate cancer in what appears to be a more progressive manner, whereas the effects of mutations in the BRCA1 gene appear to be more modest (3, 5). Therapeutically, there is no evidence to suggest which radical treatment is most appropriate for BRCA mutation carriers with localized prostate cancer. Radical treatment with surgery or radiotherapy currently appears to be clinically preferable to active surveillance. However, clinical trials are still needed to assess the most adequate management of these cases. One of the key clinical issues that need to be addressed in the treatment of BRCA-associated tumors is the choice of chemotherapy in adjuvant and palliative care. Platinum, paclitaxel and, more recently, poly(ADP-ribose) polymerase (PARP) inhibitors have been shown to be active in breast and ovarian tumors carrying germline mutations in either of the two BRCA genes, although their role in prostate cancer requires further investigation. Platinum-induced DNA cross-linking is a substrate for HR DNA repair, which is lacking in BRCA mutant cells. Therefore, these tumors show high sensitivity to platinum-based chemotherapy, both *in vitro* and *in vivo*. Previous studies in ovarian cancer have shown that mutations in both genes are associated with a similar response to platinum-based chemotherapy, and BRCA2 mutations are also associated with an increased response rate to primary platinum-based chemotherapy. Interestingly, increased genomic instability was found in BRCA2-associated tumors but not in BRCA1-deficient tumors, due to mutations or hypermethylation. This may indicate that BRCA2 lesions cause more severe HR deficits than BRCA1. In addition, PARP is an enzyme that produces large-stranded poly (ADP-ribose) from NAD. PARP is involved in the repair of single-stranded DNA breaks, and its inhibition leads to the accumulation of these damages, which may ultimately lead to replication fork stalling and DSB formation. And these DSBs are only skillfully repaired by HR. In the absence of HR, such as in the presence of BRCA mutations, these DSBs are repaired by error-prone forms of DSBs. Many clinical trials are currently investigating the efficacy of different combinations of chemotherapy and PARP inhibitors in patients with metastatic prostate cancer, either in monotherapy or in combination with other agents. In conclusion, although PARP inhibitors currently have some efficacy in BRCA-mutated breast and prostate cancers, the evidence for this remains limited (1, 4).

Notably, cases of prostate cancer in patients with BRCA2 mutations are not uncommon in clinical practice. 2023 On 12 January, a 74-year-old Chinese man was admitted to the hospital with urinary symptoms such as urinary frequency, urgency, and dysuria. His serum PSA level was 85 ng/ml, and magnetic resonance imaging (MRI) and biopsy of the prostate showed prostate cancer. At that time, he did not undergo surgery. Instead, he took enzalutamide, denosumab and the PARP inhibitor olaparib. The patient received medication for one year, after which the prostate cancer spread to the bone. The patient underwent robotic-assisted radical prostatectomy for prostate cancer and was followed up for three to six months. Of note, the patient had previously undergone a mastectomy for breast cancer. Given the patient’s family history of cancer, including his own, his grandmother’s, and his mother’s history of breast cancer, as well as her father’s unexplained death, we recommended that the patient undergo genetic screening for breast and prostate cancer. Unsurprisingly, the patient’s genetic testing revealed the presence of a BRCA2 gene mutation. Genetic testing of the patient’s relatives showed that the patient was indeed a carrier of the BRCA2:c.5987dup C(p.R1997Kfs*6) mutation. Genetic testing of the patient’s son and daughter showed that they were also carriers of the BRCA2 mutation. Notably, the variant BRCA2:c.5987dup(p.Arg1997fs) found in the patient’s son has not been reported before.

## Case presentation

**Figure 1:**
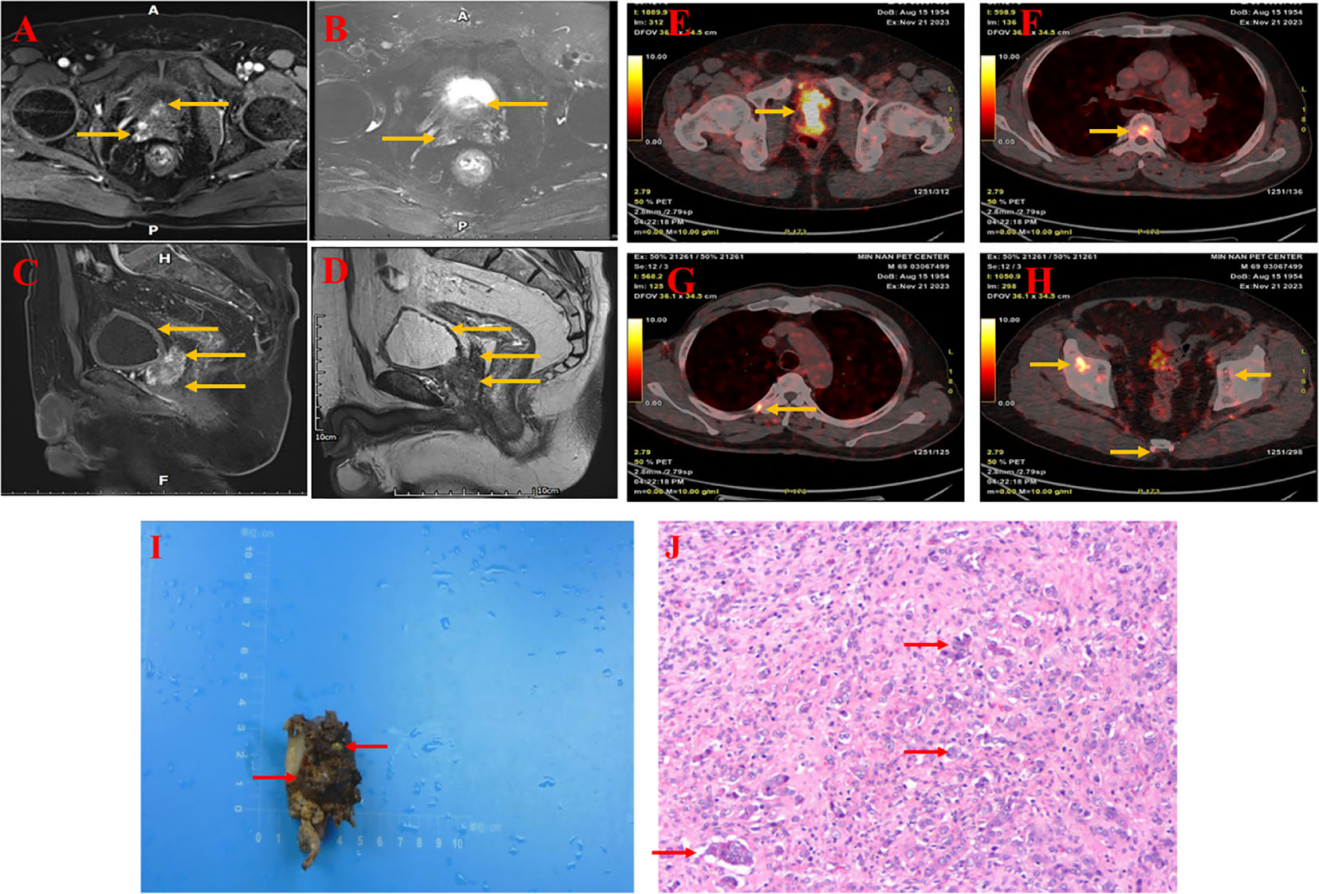
MRI of the prostate showed an enlarged prostate shape and protruding into the bladder, measuring approximately 4.1cm X 3.0cm X 3.9cm, with predominantly enlarged migratory zones and multiple prostate nodules, the larger of which was located in the right peripheral and migratory zones, with isotropic T1 and long T2 signals **(A, B)**, measuring approximately 1.1X1.0cm, and a poorly filling, slightly thickened wall of the bladder **(C, D)**. The wall of the rectal canal did not show obvious abnormal thickening. PET-CT shows malignant tumor of the prostate with seminal vesicle involvement and abnormal uptake in the prior witness **(E)**. Increased tracer uptake was seen in multiple regions of the vertebrae and ribs **(F, G)**. Abnormal uptake can be seen in multiple regions of the pelvis and sacrum **(H)**. Histology of prostate tissue carrying a BRCA2 mutation (from a prior witness). **(I)** Hematoxylin and eosin staining of formalin-fixed, paraffin-embedded tissue obtained from a radical prostatectomy in a preexisting patient shows a Gleason score of 9 (5 + 4), characterized by the prostate specimen, measuring 3.4×3.2×2.3 cm, exhibited a pale brown to dark brown hue, was solid in consistency, and displayed a partial loss of tissue, spanning 1.7×1.5×0.5 cm. **(J)** The tumor cells were arranged in a fused glandular vesicular, striated, lamellar and a few glandular duct-like arrangement, with part of the cytoplasm being pink stained, the nucleus being enlarged and vacuolated, the nuclear membrane being irregular and nucleoli being visible, and the nuclei of some of the cancer cells being fixed and deeply stained.

The patient was a 74-year-old Chinese male who was first admitted to the hospital in October 2021 with acute right-sided chest pain and a palpable right breast lump. A positron emission tomography-computed tomography (PET-CT) scan indicated a possible diagnosis of breast cancer, but no evidence of metastasis was found. A histological examination revealed a diagnosis of ductal carcinoma *in situ*, a non-invasive form of breast cancer. The patient underwent a right-sided mastectomy for breast cancer and was administered a course of anti-estrogen therapy (tamoxifen) following the procedure. Regular follow-up appointments were scheduled. One year later, the patient began experiencing urinary symptoms. The patient presented with symptoms of frequency, urgency, and difficulty in urination, as well as prolonged wait times. These symptoms were first observed on 12 January 2023, when the patient was admitted to the First Affiliated Hospital of XinJiang medical University. A rectal examination revealed an enlarged prostate gland with a hard texture and multiple palpable nodules. Laboratory tests indicated a markedly elevated level of prostate-specific antigen (PSA) at 85 ng/ml. Magnetic resonance imaging (MRI) of the prostate showed a markedly enlarged prostate accompanied by morphological irregularities. The prostate was found to be in an incomplete capsule, measuring approximately 8.85 cm x 5.25 cm x 5.02 cm. It had invaded the posterior wall of the bladder and the bilateral seminal vesicles. There was a suspicion of involvement of the rectum and the right pelvic wall, with multiple enlarged lymph nodes in the right iliac fossa and bilateral femoral vessels. Additionally, there were irregularities in the right ischium, the sacrum, and the bilateral femur. Positron emission tomography-computed tomography (PET-CT) scan revealed a malignant prostate tumor accompanied by multiple bone metastases. The prostate gland was affected, with multiple bone metastases and high metabolic activity in the lymph nodes on both sides of the pelvis. A transrectal ultrasound-guided biopsy of the prostate was then performed, with pathological examination revealing that 14 core biopsies yielded 13 cases of prostate cancer, with an average Gleason score of 8. Immunohistochemical analysis showed the presence of CK14, absence of AE1/AE3, absence of P63, presence of P504s, and PSA. The results of the aforementioned tests indicated the presence of prostate cancer (GATA3-, GCDFP15-, CD56-, Syn-). Based on these findings, the initial diagnosis was prostate cancer (T4N1M1) with bone metastasis. Given that the patient had already developed bone metastasis, surgical intervention was not performed. Instead, he took enzalutamide, denosumab and the PARP inhibitor olaparib. The patient was treated and followed up regularly. On 12 April 2023, the PSA level was found to be low, with no new bone metastases. After one year of neoadjuvant therapy, the patient returned to our institution for a PSA re-evaluation, with a stable level of 0.05 ng/ml. Magnetic resonance imaging (MRI) of the prostate showed an enlarged prostate shape and protruding into the bladder, measuring about 4.1cm X 3.0cm X 3.9cm, with predominantly enlarged migratory zones, and multiple nodules of the prostate gland, the larger of which was located in the right peripheral and migratory zones, with isotropic T1 and long T2 signals, measuring about 1.1X1.0cm, and multiple nodular short T1 and short T2 signals were seen in the right seminal vesicle glands. The bladder is poorly filled and the wall is slightly thick. The wall of the rectal canal did not show obvious abnormal thickening. (**Figures 1A–D**). PET-CT showed multiple punctate foci of increased uptake in bilateral ribs, spinal bones, pelvis, and the right upper femur, and no significant abnormalities in the remaining bones (**Figures 1E–H**). Following a detailed discussion, it was decided that the patient would undergo a robotic-assisted radical prostatectomy due to the presence of multiple bone metastases in the spine and ribs. Post-operative pathological examination of the prostate specimen revealed a volume of 3.4 cm by 3.2 cm by 2.3 cm, with a cut surface of pale brownish grey, solid, and of medium consistency, with areas of partial loss, measuring 1.7 cm by 1.5 cm by 0.5 cm. Microscopically, the tumor is observed to exhibit a fusiform, filiform, and tubular arrangement of cells, with a small number of glandular cells also present., with cytoplasmic powder staining, enlarged nuclei, vacuolar shape, irregular nuclear membrane, and nucleoli, Some cancer cells are condensed and deeply stained. Immunohistochemical analysis revealed the presence of AMACR(+),CK(+),CK7(+),GATA(+), NKX3.1(+),SYN(+) and KI67 (2%).The Gleason score was calculated to be 5 + 4 = 9. (**Figures 1I, J**). The patient was scheduled for follow-up visits at 3-6 month intervals.

**Figure 2:**
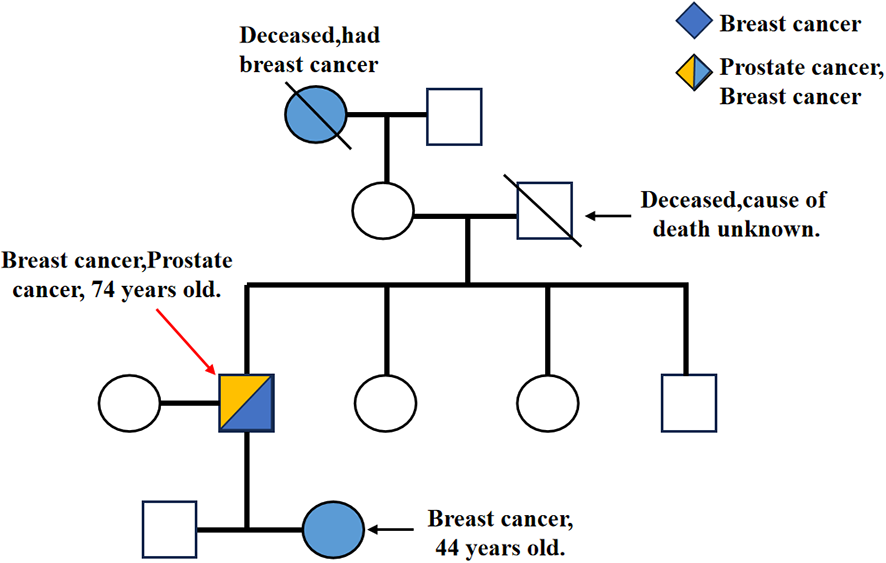
\ represents the deceased, → represents the pre-diagnosed (IIIB). IA and IVB had a history of breast cancer, and IIIB had a history of breast cancer and prostate cancer. In addition, IIIB, IVA and IVB all have different types of BRCA2 gene mutations.

Given the patient’s family history of cancer, including his own, his grandmother’s, and his mother’s history of breast cancer, as well as her father’s unexplained death, we recommended that the patient undergo genetic screening for breast and prostate cancer. We extracted total DNA from the prostate cancer tissue of the patient’s father and used it to screen for mutations in the BRCA1/2 and HRDscore genes. Ultimately, we identified a deleterious mutation in the BRCA2 gene, c.5. The 987dupC (p.R1997Kfs*6) mutation is located in the BRCA2 gene, specifically in exon 11. This mutation results in a frameshift mutation, whereby the amino acid at position 1997 is replaced by a series of other amino acids, ultimately leading to a premature termination at position 6. Additionally, the homologous recombination deficiency (HRD) score is 40, indicating a high probability of pathogenicity. The patient met the criteria for an HRD Score of 40, which is indicative of a positive result. Additionally, the patient’s daughter and son underwent genetic testing. The results demonstrated that the son’s genotype was heterozygous, and a mutation in the BRCA2 gene was identified in exon 11, specifically c.5987dupC (p.Arg1997fs). This mutation, also known as exon 11 c.5987dupC (p.Arg1997fs), is a duplication of the 1997th amino acid, which initiates a frameshift mutation. It is possible that the insertion of the new reading frame may result in the introduction of a termination codon. The premature termination codon may lead to the degradation of the mRNA, which in turn may result in the absence of protein expression. It is noteworthy that this variant has not been identified in any large population database, suggesting that it is a rare variant. In accordance with the ACMG 2015 guidelines, individuals carrying this variant are at an increased risk of developing BRCA2-related genetic disorders. The results of the genetic testing of the patient’s daughter were similar to those of the index case, with the same variant identified. A deleterious mutation, c.5987dupC (p.R1997Kfs*6), was identified in the BRCA2 gene. Unfortunately, the family members were not willing to undergo genetic testing of their grandchildren, and thus, we were unable to obtain the results of the genetic testing of the third generation. However, based on the family pedigree (**Figure 2**), it can be postulated that the family in question has a hereditary predisposition to breast cancer. In conclusion, although previous researchers have reported cases of individuals with BRCA2 mutations who developed both breast cancer and prostate cancer simultaneously, this is the first documented case of a family with a history of breast cancer who also developed prostate cancer. Furthermore, the mutation identified in the proband’s son, c.5987dupC (p.Arg1997fs), is a rare variant that has not been previously reported in large population databases. This suggests that it is a unique mutation. A case of simultaneous prostate cancer and breast cancer in an individual with a BRCA2 mutation has been reported previously, but a case of simultaneous familial breast cancer and prostate cancer has not. This is the first such case to be documented. Furthermore, the BRCA2 mutation c.5987dupC (p.Arg1997fs) in the proband’s son has not been identified in any extensive population database, indicating that it is a rare variant.

## Discussion

In this study, we report the case of a patient with a BRCA2 mutation who developed both familial breast cancer and prostate cancer. The patient received PRPP treatment for prostate cancer. Additionally, the patient’s son discovered a novel BRCA2 mutation, and his daughter developed breast cancer. A similar BRCA2 mutation was identified. Additionally, the patient exhibited prostate cancer with bone metastases. Despite the relatively brief follow-up period, the treatment regimen was found to be suboptimal, with the development of a new bone metastasis in prostate cancer within a year of initiating neoadjuvant therapy.

Prostate cancer (PCa) is the most common malignancy in men and the leading cause of cancer-related deaths. The etiology of prostate cancer is attributed to a variety of factors, with age and race being the most prominent. There is a recognized familial association between breast cancer and PCa (1–4). The role of BRCA 2 mutations in male cancers has been extensively studied, although they are present in only 1%-2% of sporadic prostate cancer cases. But,BRCA2 mutations in only 5% of PC cases. However, the Breast Cancer Linkage Coalition (BCLC) has previously reported that the relative risk of PC in men with a family of pathogenic BRCA2 mutations is 2.9-4.8, and in certain subgroups, such as those aged 65 years<, the lifetime risk is approximately 20%, with a relative risk of 7.3. More recently, the risk of PCa in BRCA1 and BRCA2 mutation carriers has been shown to increase 3.5-fold and 8.6-fold, respectively, by the age of 65 years (5–8). To date, three mutations (BRCA1 185delA, BRCA1 5382 insC, and BRCA2 6174delT) have been identified and have been studied extensively because of their relatively high prevalence. One study showed that the Korean population had the highest prevalence of BRCA2 c.7480C>T mutation, whereas the c.3109C>T mutation was more common in the Chinese population. In addition, a study in eastern Spain found that the BRCA2 mutations with high recurrence rates were c.9026_9030delATCAT, c.3264 insT, and c.8978_8991del14, which accounted for 43.2% of all mutations in the gene, with the c.9026_9030delATCAT mutation being the most common, accounting for 21.3% of BRCA2 gene mutations by 21.3%. Notably, a small sample survey in China found that among several BRCA2 gene mutation loci, the c.7397T>c locus had the highest frequency of missense mutations. With the development of genetic testing technology, such genes can be tested and the risk of developing tumors can be further assessed. In our case, genetic testing of her children showed that the patient was a carrier of the BRCA2 mutation. At the same time, the cancer history and family history of the case provided sufficient evidence to confirm that the case was indeed a familial genetic susceptibility to breast cancer (9–13). Notably, the BRCA2 gene mutation was more pronounced in prostate cancer cases diagnosed in younger individuals with more severe clinical symptoms. Although there have been previous case reports of concurrent breast and prostate cancer, it appears that there have been no reports of concurrent two cancers due to BRCA2 gene mutations.

BRCA2 is located on chromosome 13, specifically on the long arm, between 12 and 13. This gene is approximately 70kb in length and is inherited in an autosomal dominant manner. It is a tumor suppressor gene that is expressed on the surface of the cell. Mutations in this gene can result in the loss of tumor suppressor function, which can lead to DNA damage accumulation and disruption of the cell cycle checkpoint (8, 14, 15). Homologous recombination repair (HRR) is an important mechanism for the repair of DNA double-strand breaks. This process involves multiple signal transduction pathways and genes, with common pathways including BRCA1/2, ATM, CHEK2, BARD1, BRIP1, MRE11A, RAD50, RAD51C, RAD51D, and PALB2. Therefore, when BRCA function is lost, or other HRR genes are mutated or dysfunctional, it can result in a lack of HRR, leading to double-strand break repair through non-homologous end joining (NHEJ). This can cause genomic instability, resulting in the formation of genetic scars (16–18). This genomic instability may be a key factor in the predisposition to cancer in individuals with BRCA gene mutations. The absence of homologous recombination repair (HRR) due to the absence of BRCA function or other HRR gene mutations or deficiencies results in the inability to repair double-stranded DNA breaks through homologous recombination (HRR), which is termed homologous recombination deficiency (HRD). This leads to genomic instability and the formation of genomic scars, which are regions of damaged DNA. This genomic instability is believed to be the underlying cause of BRCA-associated cancer susceptibility. In this study, we evaluated the HRD status of BRCA2 in a cohort of BRCA2 mutation carriers. Despite an HRD score below 42, our findings suggest that these individuals may be at an increased risk of developing cancer. However, the presence of BRCA2 mutations has been associated with an increased risk of prostate cancer. A comprehensive evaluation of the patient revealed that he met the criteria for an HRD-positive diagnosis. It is noteworthy that DNA damage repair (DDR) pathways have recently been identified as a key factor in PCa (1, 19). The DDR system comprises multiple pathways, with one being dependent on BRCA1, BRCA2, and ATM for homologous recombination (HR). In conclusion, the role of BRCA2 in DNA repair and recombination remains inconclusive, while its functional loss This connection is inextricably linked to the repair of DNA double-strand breaks. Research on BRCA2 carriers has demonstrated a correlation between the presence of BRCA2 and an increased incidence of prostate cancer, a younger age at diagnosis, a higher prevalence of clinically significant prostate cancer, and a more aggressive disease course. This suggests that a tailored clinical approach may be necessary for BRCA 2 mutation-associated prostate cancer, similar to the personalized therapeutic approach for BRCA2-associated breast and ovarian cancers, both of which are particularly sensitive to platinum-based. This indicates that the molecular variations in patients can be used to predict their response to treatment. The response to treatment indicates that platinum-based chemotherapy is worthy of further investigation for this disease. Additionally, evidence suggests that BRCA-related prostate cancer responds to poly-ADP ribose polymerase (PARP) inhibition. In conclusion, the development of drugs targeting PARP and platinum-based chemotherapy for patients with BRCA-deficient tumors offers new therapeutic options and encourages broader BRCA testing. Research indicates that the identification of HRR gene mutations allows for the identification of an additional 10% of patients who may benefit from PARP inhibitors, while the use of HRD scores enables the expansion of this group by a further 20%. However, it should be noted that not all HRR mutations result in HRD, and therefore, not all HRR mutations are sensitive to PARP inhibitors. Currently, in prostate cancer, FDA approval has been granted for olaparib for the treatment of patients with. The case in question involved a patient with metastatic prostate cancer who had a BRCA2 mutation in the source of their DNA repair (20). However, some clinicians have also used this approach in the treatment of both sporadic and familial prostate cancer. Our patient also met the criteria for HRD positivity, which theoretically indicates a high degree of sensitivity to PARP inhibitors (olaparib) (20, 21). However, in practice, the patient’s condition did not improve despite taking olaparib for a year. In fact, the patient developed new bone metastases during the follow-up period.

In summary, we identified DNA double-strand break repair pathway BRCA2 variant in patients with both familial breast cancer and prostate cancer with bone metastases. Additionally, we discovered that the variant was present in the proband’s son, who had not been previously reported. We then proceeded to provide the proband with an individualized treatment plan, which involved the use of PARP inhibitors (olaparib). One year after initiating neoadjuvant therapy, the index case developed a new bone metastasis. He subsequently underwent a robotic-assisted radical prostatectomy for prostate cancer. In conclusion, In patients at high risk for familial prostate cancer, annual screening with serum prostate-specific antigen and rectal fingerprinting should be performed, especially in patients over 45 years of age. Genome-wide association studies (GWAS) should be performed if elevated PSA is detected. Based on the results of the BRCA2 gene test and the homologous recombination repair test, an individualized treatment plan can be formulated. Based on the results of the BRCA2 gene test and the homologous recombination repair test, an individualized treatment plan can be formulated.

## Data availability statement

The datasets used and/or analyzed during the current study are available from the corresponding author upon reasonable request.

## Ethics statement

The study was approved by the Ethics Committee of the First Affiliated Hospital of Xiamen University. Written informed consent was obtained from the patient and patient’s carers for the study and publication of this manuscript and any potentially identifiable images or data included in this article.
